# “We have a behaviour support plan, let’s have a mental health support plan”: Perspectives of staff, residents and family carers on understanding, responding to and promoting the mental health of residents within long-term care

**DOI:** 10.1177/14713012251334170

**Published:** 2025-04-14

**Authors:** Deborah Brooks, Deepa Sriram, Claire V. Burley, Rachel Brimelow, Nadeeka N. Dissanayaka

**Affiliations:** 303224 The University of Queensland Centre for Clinical Research, Australia; 303224 The University of Queensland Centre for Clinical Research, Australia; Dementia Centre of Excellence, enAble Institute, Faculty of Health Sciences, Curtin University, Australia; School of Health Sciences, 7800 University of New South Wales, Australia; 303224 University of Queensland Centre for Clinical Research, Australia; 303224 The University of Queensland Centre for Clinical Research, Australia

**Keywords:** mental health, residential aged care, dementia, nursing homes, quality indicators, qualitative study

## Abstract

Up to two thirds of people living in long-term care homes experience mental health conditions such as anxiety and depression. In addition, over half of residents have cognitive impairment or dementia. However, the quality of mental health care provided in residential care homes is often poor, and the use of mental health quality indictors is lacking. As the first step in a larger project to develop mental health quality indicators for long-term care (MHICare project), this study aimed to explore factors considered important for understanding and responding to mental health conditions and promoting mental well-being of residents. Individual interviews and small group discussions were conducted with twelve residents (both with and without dementia), eight family carers of residents with dementia, and six care home staff members, from across Australia. Qualitative data were analysed using an inductive thematic analysis approach to generate themes and a deductive approach to generate factors and map these to a Balanced Score Card quality indicator framework. Four main factors with several inter-related themes were identified: (1) Resident-related (Transitional impacts, Social connections and active engagement, and Pre-existing and co-morbid conditions); (2) Care and Communication Practices (Assessment and care strategies, Person-centred mental health care, Cultural and generational communication differences, and Staff handover and knowledge sharing); (3) Staff-related (Staff mental health awareness, Staff knowledge, practical skills and training needs, and Staff values and attitudes); and (4) Organisational (Internal staffing levels, Access to external mental health professionals, and Provision of volunteer programs). Findings will inform the key areas and initial content for mental health indicators for use in residential care homes. Once developed, these have the potential to highlight both strengths and gaps in current mental health care practices, and drive quality improvement initiatives.

## Introduction

Residents living in long-term care settings (known as residential aged care in Australia and nursing homes elsewhere), commonly experience mental health conditions. These can be complex to recognise, understand and respond to appropriately (Cations et al., 2023). Up to 58% of residents have clinically significant psychiatric needs, such as mood, anxiety, and psychotic disorders (Amare et al., 2020; Gammonley et al., 2021; Seitz et al., 2010). These rates are higher than those seen in community settings (Amare et al., 2020). Additionally, in Australia and other high-income countries, >50% of residents have a diagnosis of dementia (Australian Institute of Health and Welfare, 2023; Parveen et al., 2021). Almost all of these residents will at some point experience changed behaviours and psychological symptoms (also known as neuropsychiatric symptoms or responsive behaviours), including anxiety, depression, apathy, agitation, hallucinations and sleep disturbances (Laganà et al., 2022). The differentiation between changed behaviours and psychological symptoms of dementia and mental health conditions is complex, often presenting with overlapping symptoms (Regan, 2016). Some mental health conditions are a risk factor for developing dementia; some people with dementia develop mental health conditions (Fisher et al., 2024; Onyike, 2016; Regan, 2016; Richmond-Rakerd et al., 2022). For example, bidirectional associations between depression, cognitive decline and dementia have been demonstrated (Hayley et al., 2021; Livingston et al., 2024). Veterans with post-traumatic stress disorder (PTSD) have a high risk of developing dementia (Hutchinson et al., 2021). The prevalence of residents living with both dementia and comorbid mental health conditions (such as bipolar disorder, PTSD or schizophrenia) is unknown (Regan, 2016).

It should be noted that whilst changed behaviours and psychological symptoms of dementia is an umbrella term widely used by clinicians and health professionals, the language is controversial and may have stigmatising impacts for people living with dementia (Burley et al., 2021; Cunningham et al., 2019; Dupuis et al., 2012). There are many interacting causes of symptoms, including changes in the brain due to neurodegeneration, the interaction between the person and their social and physical environment, personal and historical factors, untreated pain and unmet needs (Dupuis et al., 2012). Burley et al. (2021) reported the viewpoints of people living with dementia and family carers on their interpretation of the causes of changed behaviours and psychological symptoms of dementia, identifying themes of decline in cognitive skills, reactive responses to changing situations, and associations with trauma memories and psychiatric history. However, clinical psychiatric symptoms may also be present and should not be discounted (Burley et al., 2021).

Subsequently, understanding and responding appropriately to neuropsychiatric symptoms is complex, with guidelines recommending a person-centred approach and non-pharmacological interventions as first-line treatment, with careful consideration of pharmacological strategies where appropriate due to the risk of serious adverse events (Dyer et al., 2018). Despite this, registered nurses working in care homes are not usually trained in the assessment and care of changed behaviours and psychological symptoms of dementia or mental health conditions (Evripidou et al., 2019; Piirainen et al., 2023; Smythe et al., 2017; Stargatt et al., 2017), even though care planning involves both physical and mental health co-morbidities (Gammonley et al., 2021). Further, resident access to specialist mental health services can be limited (Bhar et al., 2022; Perlman et al., 2019; Stargatt et al., 2017). Consequently, there have been repeated concerns about inappropriate care provision for residents, including high rates of psychotropic prescribing and use of physical restraints (Brimelow et al., 2019; McMaster et al., 2018; Walker et al., 2018). In Australia, the most common areas of substandard care are related to people living with changed behaviours and psychological symptoms of dementia and/or mental health conditions (Royal Commission into Aged Care Quality and Safety, 2021). Poor mental health care is associated with reduced quality of life for residents and higher workloads and stress for staff (Eikelboom et al., 2023).

**Figure 1.**
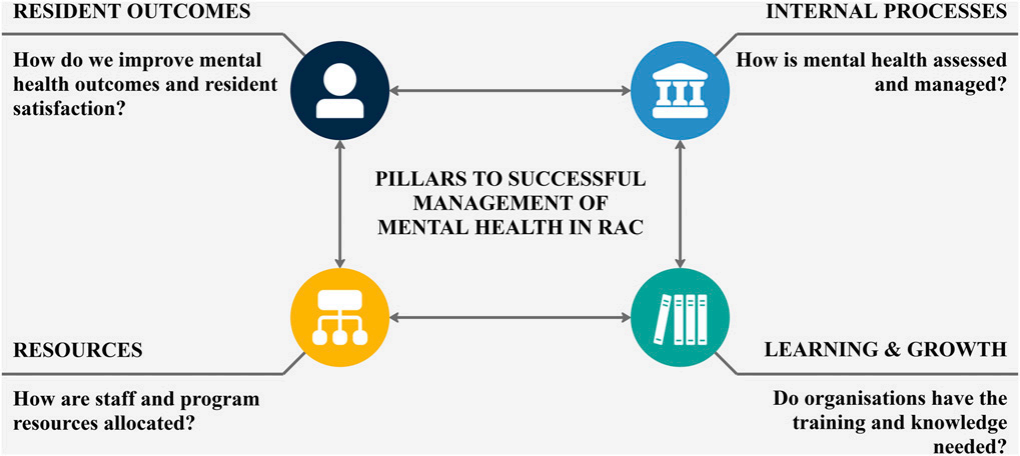
MHICare balanced score card domains.

One means of addressing inappropriate care provision is the development of quality indicators to evaluate and monitor performance and outcomes. Whilst mandatory quality indicators relating to antipsychotic prescribing and use of restraints have recently been introduced (Australian Government Department of Health and Aged Care, 2023), there is still little reporting of the mental health needs of aged care users and no standards that relate to quality monitoring of mental health care practices (Australian Institute of Health and Welfare, 2024a). To address this, we aim to develop a Mental Health Benchmarking Industry Tool for use in Residential Aged Care (MHICare Tool), to help service providers monitor and report on mental health care practices and outcomes and drive quality improvement initiatives (Brimelow et al., 2024). The MHICare Tool adopts a Balanced Score Card approach; a strategic management framework that collects and analyses information on a balanced set of perspectives and indicators (Brimelow et al., 2023). A systematic review of Balanced Score Cards used within community and hospital mental health services reported the following domains as being relevant to the residential care context; (i) internal processes, (ii) resident outcomes, (iii) learning and growth, and (iv) resources (see Figure 1).

As the first step in a larger sequential mixed methods project to develop appropriate mental health indicators (Brimelow et al., 2024), we aimed to explore factors deemed important for understanding, responding to and promoting the mental health of residents, from the perspectives of care home staff, family carers, and residents themselves. These findings will inform the key areas and content for potential mental health indicators within the four domains of the MHICare Tool.

## Research design and methods

This study utilised a qualitative descriptive design (Bradshaw et al., 2017), to gain a deeper understanding of the key factors that may influence the mental health of residents. A qualitative descriptive design is often used in health care research when the phenomenon of interest (e.g., factors influencing mental health care) is explored with participants in a particular situation (e.g. long-term care) and from a particular conceptual framework (e.g. Balanced Score Card framework). In keeping with this pragmatic approach, we conducted small focus group discussions and individual interviews in-person or online to collect data, as per participant preference and availability. The Consolidated Criteria for Reporting Qualitative Research checklist was used to report this research (Tong et al., 2007). Ethical approval for the study was obtained from The University of Queensland [HREC/2019002096].

### Sample and recruitment

Purposive criterion sampling techniques were used (Palinkas et al., 2015). Eligible participants included: (i) care home staff; (ii) family carers of residents; and (iii) permanent residents living within care homes (with or without dementia). Staff could have any direct care role, for example registered nurses, personal carer workers, recreational therapists or allied health staff. Residents needed capacity to provide informed consent (i.e. able to communicate back what participation involves after discussion with a trained researcher). Participants were eligible if they were ≥18 years, able to speak and understand English. We aimed to recruit 6–10 members from each participant group, as sufficient for theme saturation using a qualitative descriptive approach (Bradshaw et al., 2017).

The Australian ‘StepUp for Dementia Research’ registry was primarily used to recruit care home staff and family carers (Jeon et al., 2021). Study information was also posted on relevant websites. Residents were recruited via two participating homes, with the assistance of care home managers. Informed consent was gained from all participants. A researcher (CB) visited the care homes in-person to discuss the study and ensure participants had the capacity to understand what participation involved and provide informed consent before signing the consent form. There was no prior relationship between researchers and participants. Participants were offered a $50 gift voucher in acknowledgement of their time.

### Data collection

Interviews and group discussions were conducted by two female post-doctoral researchers (DS and CB) with experience in aged care and dementia qualitative research. Staff and family carers participated in individual online interviews (conducted by DS), using Zoom teleconferencing software (Zoom Video Communications). Residents participated in small in-person focus group discussions held at their care home (conducted by CB, with DS observing and taking notes). A semi-structured interview guide was developed and piloted, informed by the Balanced Score Card approach for mental health services described previously (see Table 1). A simplified version was developed to facilitate discussions for those with cognitive impairment. All participants were able to pause or reschedule the discussions at any time if they felt fatigued or distressed. Discussions lasted between 30-45 minutes, were audio-recorded with consent and transcribed verbatim for analysis. Reflective notes were also made by the two researchers.

**Table 1.** Semi-structured interview guide informed by the Balanced Score Card approach for mental health services.

1. How does mental health in aged care impact you?
2. Do you feel that current management of mental health in aged care meets your expectations?
3. How do you think practices can be improved?
4. Do you think a standardised way of reporting mental health practices across homes will help? If not why not? If yes, why?
5. The tool we are developing follows a balanced scorecard approach – this means that the tool will identify indicators across four different domains: resident outcomes, learning and growth, internal process and resources.
oWhat are some indicators of *resident outcomes* you think are important to include in the tool?
oWhat are some indicators of *learning and growth* (training and knowledge) you think are important to include in the tool?
oWhat are some indicators of *internal processes* (how the home functions) you think are important to include in the tool?
oWhat are some indicators of *resources allocation* (staff and care home resources) that you think are important to include in the tool?
6. Are there any other indicators you think are important to include?
7. What would you like to see developed from the information gathered here today?

### Data analysis

Thematic data analysis methods were utilised, informed by the research study aims and the Balanced Score Card framework to organise the data (deductive analysis), whilst allowing for emerging patterns and themes from the data (inductive analysis). Transcripts were imported into NVIVO software (Release 1.6.1) for analysis. Inductive analysis (conducted by researchers DS and DB), was used to construct the initial codes and themes and a deductive approach was used to map the generated themes onto the Balanced Score Card framework. The inductive analysis steps included: data familiarisation by reviewing transcripts, generating initial codes based on similarity of ideas, constructing themes from identified patterns, defining and refining the themes (Clarke et al., 2015; Nowell et al., 2017; Simpson et al., 2023). Frequent reflexive dialogues and meetings were held to reflect on and discuss data analysis and emergent themes throughout the data collection period and to confirm data saturation (Braun & Clarke, 2006; Thomas, 2006). There was peer debriefing and review with the research team and our consumer and community involvement group (which includes people with lived experience of dementia and long-term care), at several points as an external check of the analysis process (Bradshaw et al., 2017; Nowell et al., 2017).

## Findings

### Participants

A total of 94 volunteers (staff *n* = 25, family carers *n* = 69) were matched by ‘StepUp’ and contacted by researchers. Sixty of these did not respond after three attempts, six declined and six were ineligible. The number of participants recruited was 26 (staff *n* = 6, family carer *n* = 8, residents *n* = 12). The majority were female (staff 100%, family carer 88%, residents 83%). Geographically, participants were from Queensland (*n* = 8), New South Wales (*n* = 15), South Australia (*n* = 1), Tasmania (*n* = 1), and Northern Territory (*n* = 1). Care home staff included two recreational officers, one activities manager, one care home manager/registered nurse, one personal care worker, and one personal care worker/physiotherapist assistant. Family carers included seven daughters and one son. Four residents were living with dementia and eight without; three residents had prior experience of mental health conditions.

### Factors and themes

**Figure 2.**
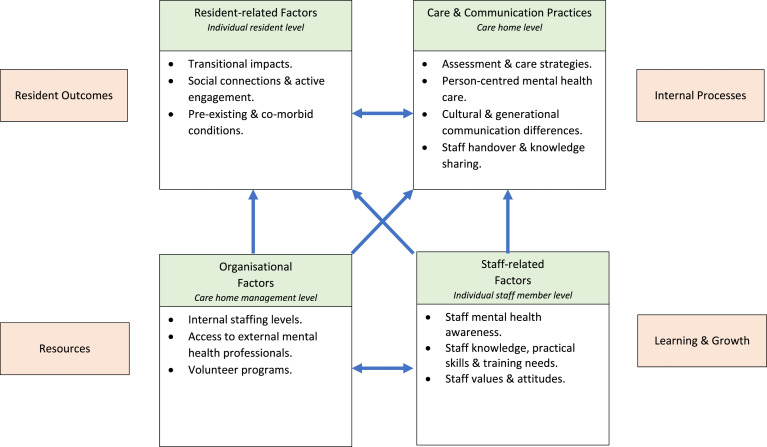
Factors and themes mapped to the Balanced Score Card domains.

As shown in Figure 2, data analysis generated a number of inter-related themes mapped within the following four main factors: (1) Resident-related (both intra- and inter-personal at an individual resident level); (2) Care and Communication Practices (at a care home level); (3) Staff-related (both intra- and inter-personal at an individual staff member level); and (4) Organisational (staff and program resources at a care home management level). Each factor and generated themes are described in turn. An additional theme relating to the development of a standardised mental health care indicator tool is also described.

#### Resident-related factors

Three themes were identified at an individual resident level (both intra and inter-personal), that impact on mental health. The first two themes, ‘Transitional impacts’ and ‘Social connections and active engagement’, were often characterised by ‘loss’ (e.g. of home and community, independence and control) and ‘lack’ (e.g. of connection to others, meaningful activities), which were further compounded by the third theme of ‘Pre-existing and co-morbid mental health conditions’, such as depression or PTSD.

##### The transition into long-term care can have a detrimental impact on a resident’s mental health

This theme encompassed both how the move is experienced by the resident (e.g., lack of choice or control), as well as the negative impacts of no longer living in their own home, such as unfamiliarity, confusion, disruption to usual routines, noise, and losing independence. Some staff and family carers described witnessing residents’ feelings of grief, distress and anger post-placement: “*And when mum was first there, she was incredibly distressed. I mean, she was distressing the other residents, and she was very angry, threatening to kill herself. It was really, really difficult*” (Family carer 05).

This difficulty of adjusting to permanent care was thought to be especially the case for someone living with dementia and/or a past traumatic experience or current mental health condition: *“I think older people often have had a number of traumatic or significant events throughout their life that also impact on how that transition goes and their relationships they form with others”* (Family carer 03). In terms of promoting more positive adjustment, it was suggested that current residents could act as a ‘buddy’ for others, to help share feelings and to settle in. It was also suggested that staff undertake a resident mental health assessment on admission.

##### Social connections and active engagement are key for mental well-being

All participants discussed the detrimental mental health impacts that living in a care home may have on residents, including sadness, depression, loneliness, and boredom. These were often interlinked and frequently perceived to be associated with a lack of relationships and social interactions with others in the care home (i.e., other residents, staff, and visitors). Some participants specifically discussed the experience of social isolation during the COVID pandemic; others talked about the residents who do not have any family or others to visit them: “*If we can have more interaction with the community and with family members, I think all of that disappears. I think for a lot of residents feeling like they’re isolated, and this has nothing to do with COVID, this is just life. Feeling like they’re isolated and away from the community makes people feel sad*” (Staff 06, Activities Manager).

Active engagement in personally meaningful activities, was also commonly raised as being instrumental to mental health. These could be sensory, social, spiritual, or physical in nature, depending on residents’ preferences. However personally meaningful activities were not always provided:

“Sometimes when I look at the activity planners, I'm not convinced that they actually take into account different people's interests… if you're trying to make sure that people are connected and engaged and have opportunities to do things that they enjoy, which is good for mental wellbeing, then it's important to know what's meaningful to people and what's available to support that” (Family carer 03).

It was also acknowledged that not all residents wished to participate in activities, and this may vary depending on how someone was feeling: *“They’ll come and knock on the door and say, ‘Would you like to?’ And I say, ‘No, I would not.’ They do, they occasionally try and persuade you”* (Resident FG1, P01). Providing reminders and a “little push to participate” whilst also respecting choice not to attend, was important to balance and discussed by both residents and staff.

##### Compounding impacts of pre-existing and co-morbid conditions

Many participants raised the complex combination of dementia, mental and physical health problems experienced by people living in long-term care. Previously diagnosed mental health conditions, in particular depression, anxiety and PTSD, as well as co-morbid physical and cognitive decline, were discussed as having a compounding impact on residents’ mental health.

“Like there might be some PTSD or something that's happened when they were younger. Say they've had a significant accident or there's something that's brought them to this mental health situation.” (Family carer 01)

#### Care and communication practices

At a service level, the following themes emerged as having an important impact upon residents’ mental health: ‘Assessment and care strategies’, ‘Person-centred mental health care’, ‘Cultural and generational communication differences’, and ‘Staff handover and knowledge sharing.’ These mostly focused on current practices and the identification of gaps where improvements could be made.

##### Current mental health assessment and care strategies

Staff talked about the signs they look for in residents, such as frustration, aggression, withdrawal, non-participation in activities, poor food intake or general demeanour. Looking for signs was deemed important, as residents who had communication difficulties due to dementia, stroke or other conditions may not be able to express how they are feeling to staff. Knowing the resident well helped staff to recognise changes in their behaviour. However, both staff and family carers acknowledged they found differentiating between mental health conditions and symptoms of dementia very difficult and that symptoms/behaviours often overlap.

“I do know one case where there was an elderly lady who'd lost her husband. She was very depressed, and she became very silent and withdrawn, and she was actually diagnosed with dementia. And it wasn't until about a year after she was in an aged care facility, and a few things changed in her life, and she became sort of a different person, and then they realised she didn't have dementia at all.” (Family carer 02)

Staff members discussed the use of assessments and tools as being useful to help them gain and share knowledge of a resident’s mental health history to inform care planning. This might include for example, using a depression assessment tool as well as talking to the resident’s GP and family to find out their mental health history. Ongoing monitoring and implementing strategies to address mental health and behavioural issues were also deemed important. This did not always happen in reality, however: “*Yes, measuring it or recording it is one thing, but then somebody’s gotta [sic] ideally do something with it because if you just know that mum’s depressed and anxious, that’s one thing. But then ideally, who then actions the outcome of that?”* (Family carer 03). The appropriate (and inappropriate) use of medication to treat mental health conditions was also acknowledged by some family carers and staff.

“Once the doctor or the neurologist changed that medication, then her mental health…she was much more calmer [sic]. So, it's a fine line of trying to get that medication right for each person. If you don't get it right, their mental health and their mental capacity can worsen very quickly.” (Family carer 01)

##### Person-centred mental health care should be central

A person-centred approach was perceived by all participants, and especially by residents, as being central to understanding, responding to and promoting mental health. For residents, having staff know them as individuals and calling them by their name made them feel like they ‘matter’. ‘Having a voice’ and being heard, was deemed especially important by residents: *“I think it’s really important that we do have a voice”* (Resident FG2, P01).

Getting to know the resident, their body language, needs, personal history, likes and dislikes, was considered imperative to inform mental health care and care planning from the perspectives of staff and family. The idea of having a specific mental health care plan for residents was also raised, in conjunction with person-centred care plans and behavioural support plans and was identified as a gap in current practice: *“We have a behaviour support plan, let’s have a mental health support plan”* (Staff 02, Care Home Manager).

##### Cultural and generational communication differences impact mental health care

To assist mental health care planning and provision, there was a perceived need for a ‘shared language’ to facilitate communication between residents and staff, who may originate from different countries. Residents with dementia may revert to their first language which may not be the same as staff, impacting communication of mental health issues. Differences in terms of how feelings are experienced and expressed were also raised, highlighting the importance of culturally appropriate mental health care: “*I was just thinking about how some of those experiences of grief, loss, life stage transitions, even anxiety, how that presents, or ideas of depression I think can differ across cultures. I’m thinking about the aged care environment and how multicultural it can be*” (Family carer 03).

Generational differences were also identified, with a perception that older people may find it difficult to talk about mental health and reach out for support.

“I think a lot of older people are used to not talking about mental health issues. It’s only nowadays that people actually will say, I need to go and see someone. They don’t feel bad about having a mental health issue. Whereas, when they were younger it was kind of a shameful thing.” (Staff 05, Personal Care Worker/Physio Assistant)

This was acknowledged by some of the residents themselves: “*Theres some days where I just feel yuck…I don’t talk. I tend to manage it myself*” (Resident FG3, P01).

##### Staff handover and knowledge sharing regarding residents’ mental health is essential

Communication between staff regarding residents’ mental health, was perceived as being extremely important but not always implemented effectively: *“I’d like a written comment by the staff re: the patients, so that it's read by the next person coming on or when they change staff*” (Resident FG1, P03). This was discussed mainly in the context of staff shift handovers or handovers to newly appointed staff, and knowledge sharing of strategies to support mental health or changed behaviours and psychological symptoms of dementia: “*Information that’s passed, or passed on from one shift to the next, so that we could address the needs and definitely the mental health of our residents*” (Staff 04, Personal Care Worker).

#### Staff-related factors

At an individual staff member level, three inter-related themes relating to ‘Staff mental health awareness’, ‘Staff knowledge, practical skills and training needs’, and ‘Staff values and attitudes’ were identified as key to understanding and responding to residents’ mental health. The first of these were characterised by a current lack of and need for improved mental health awareness and training for residential care staff.

##### Need to improve staff mental health awareness

Family carers consistently identified a lack of staff awareness of mental health issues in their residents. Some staff agreed that there was a need for more focus on this in long-term care, especially with a perceived increase in residents living with both mental health conditions and dementia.

“It’s probably something that hasn’t really been a focus of in aged care before, but we’re getting so many people with younger onset dementia, with mental health conditions with combined dementia and mental health conditions, that it is an area that we really need to start to focus on and upskill in.” (Staff 02, Care Home Manager)

##### Improved training in mental health and dementia is required to improve staff knowledge and skills

This was identified as important by all participants. Family carers were concerned that staff had received minimal training in this regard, and that a lack of knowledge and skills impacted on the care residents received: *“Some of them weren’t very well trained in dementia as such. When it comes to managing their mental health and capacity, their training was very minimal”* (Family carer, 01). Staff agreed that knowledge of mental health issues when working with residents was important but that they had not received adequate formal training for this.

“I think we need more training, need more formal training. We do a lot of training on dementia obviously being in aged care, we need to know a lot about that. But we don't do any as such on mental health.” (Staff 06, Activities Manager)

Some residents emphasised the importance of practical experience over theoretical learning, however: *“I think practice is more important than anything. I think more practice and less bookwork is more important”* (Resident Interview, 01).

##### Staff values and attitudes towards residents are important in promoting mental wellbeing

For residents this was the most important issue they discussed and related to the care home employing staff with the required person-centred qualities, such as being approachable, cheerful, patient and compassionate: *“They really are very kind, very caring, got a good sense of humour, all the qualities that I think that you need”* (Resident FG3, P02). Staff compassion and sensitivity when working with residents living with dementia and/or mental health conditions was also highlighted by family members and staff: *“The touch that has compassion, that here I am, I’m walking with you, I know how you feel”* (Staff 01, Recreational Officer).

Trust and respect were similarly valued. For one resident, this meant being able to trust and confide in a staff member about their feelings and emotional well-being.

“Being able to feel that you can speak to the staff privately, and they’re not taking it outside of the conversation you’ve had. Because from my experience and other people, we need to have an outlet.” (Resident FG2, P01)

#### Organisational factors

At a care home management level, themes included ‘Internal staffing levels’, ‘Access to external mental health professionals’, and ‘Provision of volunteer programs.’ The first two themes were characterised as being current barriers to caring for resident mental health, whilst the latter was a potential facilitator for promoting mental well-being.

##### Internal staffing issues are a barrier to responding to mental health needs

The umbrella term ‘staffing issues’, included high staff turnover, under-staffing, and time pressures on staff to complete care tasks. These issues were discussed by all participants and meant that staff were not able to spend the time they needed to focus on or implement strategies to improve residents’ mental health.

“But they don't focus on it [mental health] because there are times that the residential facility is overworked. There's no staff. They don't have the time to talk about it. There's [sic] more cases that needs to be done rather than focusing on that mental health strategy of the person.” (Staff 02, Care Home Manager)

##### Access to external mental health professionals is needed

In the light of internal care home staffing issues and lack of training, access to external mental health professionals, such as geriatricians, psychiatrists, and psychologists, was deemed important for ensuring good mental health care for residents. However, this was often not the experience of the participants.

“The whole time I was there I don’t think I ever heard anyone talking about mental health. There was [sic] certainly no visits from mental health professionals, counsellors.” (Family carer 04)

##### The provision of volunteer programs may facilitate resident mental well-being

This was suggested by some family carers as a means of mitigating staff shortages and providing more social and emotional support to residents who don’t have frequent or any visitors. Some family carers were already doing this (informally) with other residents when they went in to visit their family member or wanted to become a volunteer themselves.

“I'm actually considering asking the nursing home if I can just be a visitor and come and spend some time with those who don’t get visitors. You’ll just see them, they’re clearly bored, that can impact on their behaviour sometimes.” (Family carer 04)

### Perceptions of a standardised mental health benchmarking tool

Many of the staff and family carers were supportive of a tool that may help to improve practices and mental health outcomes for residents. However, some noted it would be hard to standardise such a tool, and that some flexibility would be required: “*I think having a standard practice is good, but then there’d need to be flexibility. With mental health, it’s such a hard area to target really, isn’t it? Because everybody’s different. … I think it’d be hard to streamline it, but it would be good if you had everybody on the same page, and the processes on the same page*” (Family carer 01). Staff were also wary about the workload such a tool might add to busy schedules. A tool that was not user-friendly or too time-consuming would be a challenge for implementation.

## Discussion

The development of mental health quality indicators (including structure, process and outcome indicators) in residential care is still lagging behind those for hospital and community-based services, and tends to focus only on depressive symptoms, psychotropic prescribing and physical restraints (Australian Government Department of Health and Aged Care, 2023, OECD/European Union, 2013). As part of an innovative study to develop a Mental Health Benchmarking Industry Tool for Residential Aged Care, we identified a number of key areas that impact understanding of and response to residents’ mental health and well-being, from the perspectives of care home staff, residents and family carers. These findings provide insights into current mental health practices in long-term care, barriers to the provision of quality mental health care and potential areas for improvement that can inform the development of mental health quality indicators. Novel findings relate to the development of mental health care plans by care home staff, improved staff training, and attitudes towards a mental health benchmarking tool for use within residential care.

At a resident level, the transition into residential care was identified as a risk factor for poor mental health, particularly in the context of dementia and/or pre-existing mental health conditions. This is referred to as ‘relocation stress’, where care home placement is associated with increased anxiety and depression in older adults (Polacsek & Woolford, 2022). Admission processes therefore provide an opportunity for improved mental health assessment and care planning by staff. Whilst strategies to support the mental health of residents following admission were suggested by some participants (e.g., ‘buddy’ programs), there is little evidence of the effectiveness of these to date and further research on such strategies is needed (Polacsek & Woolford, 2022).

As reported in our study, low levels of engagement in personally meaningful activity within care homes negatively impacts mental health (Davison et al., 2022). However, such activities are not consistently provided and a balance is needed between staff encouragement and residents having the choice to participate or not. Documenting and monitoring the provision of, and engagement with, personally tailored activities may therefore provide an opportunity for quality improvement. Volunteer programs were also suggested to provide improved social and emotional support to residents, however evidence for their effectiveness on resident mental health outcomes (e.g., loneliness, depression, anxiety) is currently lacking (Handley et al., 2022).

In terms of care practices, the use of formal assessment tools (e.g., for depression) and physical health screens (e.g., bowel movements, hydration), as well as knowledge of a resident’s mental health history, is needed to inform appropriate care planning upon change in health status. The use of validated mental health assessment and screening tools can be evaluated at a care home level. However, this is also dependent on having a qualified staff member available to make such assessments. There was little discussion of mental health treatment beyond the use of medication, and the under-use of non-pharmacological mental health interventions for residents has been previously reported (Cations et al., 2022; Stargatt et al., 2017). However, this could be addressed by the suggested mental health care plans for residents (separate to the Behaviour Support Plans that relate to restrictive practices in aged care, mandated by the Australian government in 2021). In Australia, mental health care plans are not normally developed by care home staff, but by a general practitioner who provides or refers the person for services under the government-subsidised ‘Better Access to Mental Health Initiative.” However, most residents are not eligible for these subsidised services, and neither they nor their families are likely to afford them (Stargatt et al., 2017). Indeed, a recent study found that less than 3% of residents with a mental health condition accessed subsidies for mental health services under this scheme (Cations et al., 2022). A mental health care plan developed by care home staff (in conjunction with the resident, their family and other health professionals as appropriate), would therefore provide an opportunity for better mental health care within this setting.

Whilst person-centred mental health care was promoted by all participants, barriers to providing such care related to low internal staff levels, high staff turnover, and poor staff attitudes; a finding consistent with the wider literature (Batchelor et al., 2020). Staffing issues often mean that the focus is on physical and task-oriented care rather than psychological care (Kuo et al., 2019). Very few care homes directly employ psychologists, however occupational and diversional (or recreational) therapists are more commonly employed and can offer opportunities for addressing mental well-being via activity programs (Stargatt et al., 2017). Such staff are unlikely to be trained in assessing for mood disorders such as anxiety and depression though, nor in the delivery of evidence-based treatments for neuropsychiatric symptoms of dementia or mental health conditions (Cations et al., 2022). The attitudes of direct care staff were identified as most important by residents in promoting their mental well-being on a day-to-day basis, but international studies suggest that less than 50% of residents have a ‘friendly conversation with staff’ most or all of the time (Morris et al., 2018).

Many of these perceived barriers were linked to a need for improved staff training in mental health awareness, assessment and care, which is typically absent (Muralidharan et al., 2019). For example, there is currently no mandatory dementia care or mental health training for long-term care staff in Australia or the UK (Alzheimer’s Society, 2024; Australian Government Department of Health, 2020). A lack of staff training in detecting anxiety and depression has previously been identified as a barrier to accessing psychological treatment services for residents (Stargatt et al., 2017). Improved training is likely to reduce mental health stigma among staff, improve communication and increase referrals to external mental health services (Muralidharan et al., 2019). However, the availability of such services is limited not only in terms of funding but also the availability of qualified practitioners such as psychologists and counsellors, who specialise in working with older adults (Perlman et al., 2019; Stargatt et al., 2017).

Cultural barriers to appropriate mental health care were also identified. Australia, like many developed countries, is a multi-cultural society and an estimated 20% of residents are from culturally and linguistically diverse (CALD) backgrounds (Administration on Aging, 2020, Australian Institute of Health and Welfare, 2024; National Academies of Sciences Engineering, 2022). Additionally, over 30% of nursing and caring staff have identified as being from a CALD background, however there is little overlap between the cultural backgrounds of residents and staff (Australian Institute of Health and Welfare, 2024b; Sheehy et al., 2024). As a result, language differences (compounded by lack of access to interpreters), and culturally inappropriate care, have been identified as issues that still need to be addressed (Gaviola et al., 2024). Cultural differences in the conceptualisation of mental illness, as well as generational attitudes and stigma towards discussing and seeking help for mental health issues, are barriers to mental health care for people from CALD communities and older people more broadly (Elshaikh et al., 2023; Gilbert et al., 2022; Radhamony et al., 2023).

Communication between care home staff (e.g., at shift-to-shift handovers), about residents’ mental health status, any changed behaviours and strategies being successfully utilised or otherwise, was identified as a key area for improvement. There is little literature about the quality and content of such handovers within residential care compared to hospital settings (Poelen et al., 2023), however influencing factors include “workplace culture, shift patterns and the extent to which they involve all those on duty or just those with professional qualifications” (Moriarty et al., 2019). Audits of nursing documentation have been suggested as a means of improving quality, and could be implemented within this setting (Tuinman et al., 2017).

Finally, most participants were in favour of a benchmarking tool for mental health care in long-term care, with caveats about ease of use and not being too time-consuming for staff to utilise. Adjustment (flexibility) for case-mix and other service variables was deemed necessary. Internationally, benchmarking initiatives for mental health services are more commonly found in community and in-patient settings, with less development in residential care settings (Brimelow et al., 2023; Samartzis & Talias, 2020). The proposed MHICare Tool will use the Balanced Score Card approach that focuses on structure (care home policies, staff training), process (assessment, care strategies) and outcome quality indicators at a care home level.

## Strengths and limitations

Few studies have engaged care home staff, residents and families to understand factors important to them for understanding and responding to mental health in long-term care settings. Engaging with key stakeholder groups is imperative to ensure the proposed MHICare Tool is relevant and reflects current practices and desired outcomes. We acknowledge that this qualitative study involved limited numbers of care home staff, however diverse roles were included. Similarly, family carers were mostly daughters and no spouses/partners were recruited. However, recruitment continued until no new themes were identified to confirm data saturation. Residents were recruited from two care homes and may not be representative of residents in other care homes. Gender balance was not achieved, due to the greater proportions of care home staff, family carers and residents who identify as female. Despite these limitations, rich and varied qualitative data were obtained to identify areas of importance from a range of perspectives, including residents with dementia who are often excluded from such studies.

## Conclusions

This paper reports on the first stage of a mixed methods co-design process to identify key areas of need and appropriate content for a new Mental Health Benchmarking Industry Tool for Residential Aged Care. This tool has the potential to be used across Australia and internationally, highlighting both strengths and gaps in mental health care and driving quality improvement initiatives.

## Data Availability

On reasonable request and as per participant consent. *
